# Self-interest, compassion, and consistency in an environmental ethics class: would students give up their retirement to stop the coronavirus?

**DOI:** 10.1007/s40889-021-00126-2

**Published:** 2021-07-12

**Authors:** Emily A. Davis, Thomas P. Wilson, Bradley R. Reynolds

**Affiliations:** Department of Biology, Geology, and Environmental Science, University of Tennessee at Chattanooga, #2653, 215 Holt Hall, 615 McCallie Ave, Chattanooga, TN 37403 USA

**Keywords:** Environmental ethics, Future generations, Egoism, Compassion, Altruism, Consistency

## Abstract

During spring of 2020, environmental ethics students at a medium sized metropolitan university in the Southeastern United States were asked to read and comment on classic essays from Robert Heilbroner and Garrett Hardin, essays regarding our responsibilities towards future generations. In general, students seemed to hold more with Heilbroner’s stance, which left room for compassion, while condemning Hardin’s harshness. Students were then asked to provide written responses stating whether they would personally sacrifice their eventual retirement in order to stop COVID-19 and the reasons for their views. Responses were analyzed and categorized to detect inconsistencies between students’ described views and their willingness to personally sacrifice for the sake of others. Almost 72% of respondents asserted that they would be willing to intervene to stop the novel coronavirus by sacrificing their retirement. A fair number of respondents that stated they would sacrifice (28.6%) said that they would do so because they would benefit personally from the avoidance of guilt and/or from the opportunity to feel good about themselves, suggesting that even seemingly selfless behaviors are sometimes driven by egoistic motivations. Forty percent of all respondents held inconsistent views. Most notably, a number of students condemned Hardin for his lack of compassion, yet were not willing to act compassionately themselves.

## Introduction

According to Des Jardin (2013), applying ethics “involves a self-conscious stepping back … to reflect on what type of life we should live, how we should act, and what kind of people we should be” (p. 6). During the spring semester of 2020, an upper-level lecture-based environmental ethics class entitled Values and the Environment with 48 students met three days a week at a medium-sized metropolitan university in the Southeastern United States. The students, in a manner consistent with Des Jardin (2013), were challenged to step back and reflect. Students listened to lectures, engaged in debates and class discussions, participated in in-class writing assignments, and completed assigned readings. As part of the class’s consideration of the present generation’s responsibilities to future generations, two of the assigned readings were Garrett Hardin’s “Lifeboat Ethics: The Case Against Helping the Poor” (1974) and Robert Heilbroner’s “What Has Posterity Ever Done for Me” (1975), classic essays that are often included in environmental ethics anthologies (Pojman 2005).

In “Lifeboat Ethics: The Case Against Helping the Poor,” Hardin (1974) argues that wealthy nations such as the United States are metaphorical “lifeboats” with a limited capacity, and that the poorer nations and their people are in the water, desperately wanting in the lifeboat, in order to escape drowning in the open sea. Helping the struggling, starving populations of poorer nations, out of either Christian charity or Marxist obligation (Hardin 1974), sounds morally appealing to many. Hardin however argues that doing so would only serve to drain the resources of wealthy nations and that the overpopulation that would result would in fact ultimately hurt humankind, the environment, and posterity. While helping those in poorer nations may initially appear benevolent and appropriate, it is actually in the long term detrimental to both the poorer nations receiving aid and to the rich nations rendering aid. Therefore, rich nations should not help poorer nations, which typically have a much faster population growth rate. According to Hardin, helping those in poorer nations survive only contributes to overpopulation, subsequent resource depletion, habitat loss, environmental degradation, and suffering. In short, those in the metaphorical lifeboat should not let others aboard. Otherwise, they risk overburdening resources and sinking the lifeboat altogether (Hardin 1974). Hardin is willing to sacrifice some present lives as a means of ensuring a better quality of life for those that will exist in the future. This is, Hardin (1974) admits, “harsh,” but in his view necessary.

Robert Heilbroner (1975) is also mindful of future generations in “What Has Posterity Ever Done for Me?”. Many seemingly “harsh” arguments concerning posterity come from a rational, logical standpoint, but no amount of rationality can make people care about future generations (Heilbroner 1975). In his call for something other than harsh rationality, Heilbroner leaves some room for compassion. While the human race likely cannot survive indefinitely, and while it is difficult to imagine the lives of future generations, it could be argued that the current generationhas a moral obligation to not only extend the survival of our species, but to be at least somewhat compassionate in the process. Such compassion arises from the principle of sentiment or inner-conscience (Heilbroner 1975). At the very least the current generation has a moral obligation to exercise personal responsibility as we go about making decisions that will potentially impact other people (Heilbroner 1975).

Heilbroner (1975) acknowledges that it is human nature to care most deeply about problems that affect us personally and immediately, no matter how seemingly insignificant compared to larger catastrophes, given that we are creatures of self-interest. Drawing upon economist Adam Smith (1759, quoted in Heilbroner 1975), he uses the hypothetical example of a “man of humanity” learning of a massive earthquake that has swallowed the entire population of China. Perhaps this man also learns that tomorrow he is to lose his little finger. This man has little reaction to the catastrophic earthquake, an event that does not personally affect him. However, after learning about the imminent loss of his finger, a less significant but more personal problem, the man is distraught. Given the choice then, would this man prefer the destruction of an entire population or the loss of his smallest finger? Would this man voluntarily give up his finger to save China? He perhaps would, if he was truly responsible for all of those innocent lives, even though to give up his finger flies in the face of self-interest (Heilbroner 1975). Heilbroner comes to the following conclusion: there are people who would perhaps prefer a catastrophic event happen to others rather than to give up their own personal comforts, but these individuals might feel differently if they actually had to take personal responsibility for their decision.

Hardin (1974), in a logical, coherent, rational manner, says be harsh if need be, in order to ensure the survival of future generations. Heilbroner says to be mindful and protective of humanity/posterity, even though to do so may not be entirely rational. Is it to be rational self-interest or altruistic compassion when it comes to “matters of life and death?” This is the choice before us.

## Materials and methods

Students were asked to read the Hardin and Heilbroner essays and to then come to class prepared to discuss (on 2-14-2020). Students were asked to self-reflect and respond to the following in writing during class: is Hardin correct in his claim that by helping poorer countries with increasing populations we act immorally (from Pojman’s (2005) Environmental Ethics: Readings in Theory and Application)? Students were likewise asked during class to respond in writing to the following: would you give up, not your finger, but your retirement (social security, investments, savings, and inheritance) to stop the coronavirus in China and prevent a possible global pandemic? Why or why not? Students were given approximately 25 min to compose their answers. The responses were then analyzed and classified into categories. Two students were then purposively selected out of the larger group for structured interviews to more fully explore emerging themes: both interviewees were environmental science major in their early twenties.

In terms of the limitations of the study, the participating students and their responses were from a single course during a single semester at a single university in the southeastern United States. Additionally, during this single semester, the instructor and the students, because of Covid-19 and in the interest of safety, were forced to transition from a face-to-face modality to an online modality. This represents an unplanned restructuring of the learning environment that may have influenced both the instructor and student responses.

## Literature review

Much of the literature regarding altruism and self-interest relies on philosophical reasoning, as well as on empirical studies from the field of psychology. Additionally, experts on the subject are in disagreement about what truly motivates human behavior in this regard, and arguments for one view often mirror arguments for the opposing view (Glasgow 1978; Rosas 2002; Steen 2011).

It has been argued that all decisions made by humans, whether consciously or unconsciously, are made to benefit self, even when the actions are seemingly compassionate towards others. These actions are driven by psychological egoism, the notion that all humans are ultimately motivated by self-interest (Slote 1964; Glasgow 1978; Sober and Wilson 1999; Mercer 2001; Hobbes 2018). A popular argument is that there is an evolutionary benefit for an individual to act in seemingly altruistic and helpful ways (Chudek et al. 2013; Piccinini and Schulz 2018). For example, helping someone might make it more likely that the person being helped reciprocates in the future, thereby increasing the likelihood of the helper’s survival or reproductive success, which is an egoistically produced desire (Piccinini and Schulz 2018). Another branch of psychological egoism is “psychological hedonism,” which states that actions are not made for one’s survival, but rather for one’s pleasure or avoidance of pain (Griffiths 1991; Sober and Wilson 1999). The terms ‘pleasure’ and ‘pain’ have been broadly interpreted, and thinkers have stated nearly all situations could potentially fit into these two categories (Garson 2016).

In contrast, some philosophers and social psychologists have disputed the idea that individuals only act out of self-interest and argue true altruism does in fact exist (Monroe et al. 1990; Batson and Shaw 1991; Rosas 2002; Clavien 2012; May 2011a; May 2011b). A notable study by Monroe et al. (1990) found that some rescuers of Jews in Nazi Germany put others first even when they were personally struggling, and that they did not rescue people to feel good about themselves or benefit in any way, and even felt embarrassed when praised. Some argue that it is in many cases “simply too cognitively demanding and hence unfeasible” to process all potential outcomes (Piccinini and Schulz 2018), so individuals do not make such conscious calculations about when to be compassionate for their own gain (Monroe et al. 1990). In other words, there are too many potential variables in any given situation that influence whether helping another individual will be directly beneficial to the person giving the help, which may be why some individuals do not consistently act in altruistic or egoistic ways in every situation.

## Results – Reflections

During the class discussion, students were seemingly appalled and even outraged by Hardin’s stance. The more vocal students condemned Hardin for his harsh views. Students seemed to be more accepting of Heilbroner’s work, perhaps because Heilbroner allows for some notion of mercy and compassion and sentiment, thereby allowing for “the grandeur and dignity, and superiority of our own characters” (Smith 1759, quoted in Heilbroner 1975). During the spring of 2020, COVID-19 had taken hold in China and a pandemic seemed imminent, although at this point the virus had not fully hit the US. How many people were set to die in the present and in the future, both in China and throughout the rest of the world?

Students were classified into five different groups based on their responses to the aforementioned questions:

- Group 1) Students that Refused to Take a Stance.
- Group 2) Students that Criticized/Condemned Hardin and Were Willing to Compassionately Intervene/Sacrifice.
- Group 3) Students that Criticized/Condemned Hardin and Were Not Willing to Compassionately Intervene/Sacrifice.
- Group 4) Students that Agreed with Hardin and Were Willing to Compassionately Intervene/Sacrifice.
- Group 5) Students that Agreed with Hardin and Were Not Willing to Compassionately Intervene/Sacrifice.

A description of each group along with an explication of recurring themes follows, with pronouns made plural to mask genders:

Group 1 (Representing 7.5% of 40 Respondents) - Students that Refused to Take a Stance: This group, consisting of three individuals, refused to take a firm stance on one or both of the questions. All refused to take a hard stance on Hardin’s argument. When asked if they would sacrifice their retirement to stop the coronavirus, one individual refused to take a stance. A second individual indicated they would not sacrifice their retirement, claiming another virus was bound to happen anyway, and a third individual indicated that they would sacrifice any future retirement in the hopes that such a sacrifice would produce a “better afterlife.”

Group 2 (Representing 40.0% of 40 Respondents) - Students that Criticized/Condemned Hardin and Were Willing to Compassionately Intervene/Sacrifice: This group, consisting of 16 individuals, showed several repeating themes. The most common theme among this group, with 37.5% of respondents making this argument, was the claim that providing some forms of aid (e.g., contraceptives, education, etc.) would actually reduce the populations of poorer nations, not increase them. The second most common theme, with 31.3% of respondents giving this statement, was the claim that they would sacrifice their retirement because the value of their retirement would not be very much anyway, or that they otherwise were not depending on their retirement, potentially highlighting a myopic view of the world in reference to themselves.

Group 3 (Representing 12.5% of 40 Respondents) - Students that Criticized/Condemned Hardin and Were Not Willing to Compassionately Intervene/Sacrifice: This group, consisting of 5 individuals, held inconsistent views. They condemned Hardin for his lack of compassion, yet were not willing to be compassionate themselves. Four repeating themes appeared in 40% of the responses. These themes were showing remorse when saying they would not sacrifice (“I feel horrible admitting this,” etc.), claiming another virus is bound to come along anyway, recognizing Hardin as rational, even if they do not agree with him, and claiming that providing some forms of aid, such as contraceptives and education, would actually reduce rather than increase the populations of poorer nations.

Group 4 (Representing 27.5% of 40 Respondents) - Students that Agreed with Hardin and Were Willing to Compassionately Intervene/Sacrifice: This group, consisting of 11 individuals, held views that recognize a rational, logical argument, yet still allow for compassionate intervention. This group held inconsistent views in the way that they agreed with Hardin’s harsh views, yet were willing to personally sacrifice for others. The two most common repeating themes, with each statement appearing in 36.4% of the responses, was the assertion that the respondents would sacrifice partly in order to avoid guilt or otherwise gain a sense of fulfillment, and the claim that they were already planning to work through retirement or would not mind working instead of retiring.

Group 5 (Representing 12.5% of 40 Respondents) - Students that Agreed with Hardin and Were Not Willing to Compassionately Intervene/Sacrifice: This group, consisting of five individuals, held rational yet harsh views. The most common repeating theme among this group, with 60% of respondents giving this statement, was the claim that the virus is a positive as far as addressing overpopulation. Eighty percent of respondents in this group also gave a unique statement in their answer, a statement that did not repeat among other respondents.

Across the five groups, the most common repeating theme, with 55% of all respondents giving this statement, was the determination that Hardin is rational in his argument. This recognition was seen across multiple groups, among those who both agreed and disagreed with Hardin. The second most common theme, with 30% of all respondents giving this statement, is the claim that providing some forms of aid, such as contraceptives and education, would actually reduce the populations of poorer nations, not increase them.

Some additional interesting statements among several individuals were present as well. Two students expressed a rather disturbing anti-Chinese sentiment, claiming that they believe China’s government is corrupt and that China is using the virus as a weapon; therefore, a reduction in China’s population would be a positive. Two individuals claimed they would be more comfortable sacrificing only a portion of their retirement and not all. Interestingly, one individual argued against Hardin’s views by claiming the way the “lifeboat’ will eventually sink is in how rich countries treat the environment and not because of the influence of poorer countries. One individual claimed they would not be willing to sacrifice until the virus turned into a true pandemic.

## Results – Interviews

Two students were purposively selected out of the larger group for structured interviews to more fully explore emerging themes: both were willing to participate and were environmental science majors in their early twenties. Summaries of the interviews follow, with pronouns again made plural to mask genders:

Interviewee 1 first pointed out that even though Heibroner admits that his views are not rooted in logic, he is still the correct one, more so than Hardin. Interviewee 1 said that they fundamentally agreed with Heilbroner. Yet, they insisted that they would not give up their retirement to stop the coronavirus, seemingly contradicting Heilbroner’s call for personal responsibility. While the student seemed to champion the need for compassion and insisted that their own views were the same as Heilbroner’s, they still chose to follow along with self-interest rather than allow room for compassion in the hypothetical situation. Recognizing that giving up their retirement would “affect my ability to sustain myself when I’m older,” they insisted that they would not give up their retirement to stop the virus. They did suggest that they would *perhaps* give up a portion of their retirement, but not all of it. Interestingly enough, Interviewee 1 recognized that they were contradicting themselves and going against their own endorsement of Heilbroner and his call for compassion. The student was then given the following quote from the Stoic philosopher Epictetus and asked to comment on it: “to live a life of virtue, you have to become consistent, even when it isn’t convenient, comfortable, or easy.” Interviewee 1 insisted that they agreed with the quote, even though their overall stance again was contrary to it. Interviewee 1 said that while Epictetus’s assertion “sounds good on paper … when it really comes down to doing these actions, it’s really hard to stick to that.”

Interviewee 2 asserted that they agree completely with Heilbroner and that they hold the same views. The student never went so far as to say Hardin was “wrong.” Instead, Interviewee 2 acknowledged that Hardin brings up “good points … [and] pressing issues about overpopulation that we still need to answer.” The student then said that they would willingly give up their retirement to stop the coronavirus. They said “I think about putting myself in the shoes of others, and I would want to stop [the virus from] affecting [others]. I don’t need money or savings, just the things I am passionate about and my family … I want to ensure other people can have these things as well.” Interviewee 2 did admit that they would be less likely to give up their retirement, if they were older. They agreed with the Epictetus quote about consistency and asserted the importance of “practicing what you preach,” but also pointed out that people contradict themselves all the time. Interviewee 2 did not condemn such people, but rather stressed that to contradict oneself is “not a shortcoming … [it’s] just human nature.”

## Discussion

Most students (28 of the 39 respondents who took a stance, 71.8%) asserted that they would be willing to intervene and stop the novel coronavirus by sacrificing their retirement. However, the number of individuals who reported that they would *not* be willing to sacrifice (the other 28.2%) cannot be overlooked. How could the respondents, all students studying environmental ethics, have such opposing views, views supported by such a wide variety of “reasons?”

Some of the students were harsh and self-centered in their views. These students, in wanting to hold on to their retirement, were clearly motivated by self-interest and could therefore best be described as psychological egoists (Des Jardin 2013). What about the other students that said that they wanted to help and were willing to give up their retirement to do so? Were these students (the 71.8%) truly classical psychological altruists, looking to help others for the sake of the ones receiving the help (Piccinini and Schulz 2018)? Or was psychological egoism the driving factor here as well, with the self-interest simply expressing itself in a different way? Those who stated they would not sacrifice would clearly benefit from keeping their retirement. But those who stated that they would sacrifice would benefit as well. They would benefit through the avoidance of guilt and from the opportunity to feel good about themselves. This was reflected in the data. Eight of the 28 respondents (28.6%) who stated they would sacrifice said that they would do so because they would feel guilty if they did not, or that they would otherwise gain some sense of fulfillment in sacrificing. Clearly, these individuals were just as concerned with “self” as the students who wanted to hold on to their retirement for purely financial reasons. These students could perhaps be classified as nonclassical psychological altruists, in that they look to help others at least on some level for their own sake, more so than for the sake of the individuals receiving the help (Piccinini and Schulz 2018). In wading through the motivations associated with any helping behavior, two questions must always be considered: first, what is the ultimate desire of the individual providing help and second, how is the ultimate desire produced: egoistically or altruistically (Piccinini and Schulz 2018)?

Whatever their motivation, in this study, respondents were given time to reflect. They were given time to construct an answer concerning a hypothetical situation, unlike many real-world scenarios with real consequences, where a decision must be made on the spot. They had the opportunity to overcome, at least somewhat, the aforementioned unfeasible cognitive demand associated with processing all of the potential outcomes. It is likely that each individual thought of several different variables and of potential consequences associated with their decisions, and then placed different values on those variables and consequences. Powers and Vogel (1980) would call this moral identification and ordering, which is “the ability to identify important issues, determine priorities, and sort out competing values” (Johnson 2005, p. 236). In short, the Values and the Environment students likely performed a cost-benefit analysis and weighed the costs against the benefits (Des Jardin 2013). Such reasoning was apparent in the aforementioned interviews, with Interviewee 1 and Interviewee 2 both weighing different factors as far as giving up their retirement (the whole retirement versus a portion of it, age at which they would have to give it up, etc.).

A tendency to fall back on a cost-benefit approach could explain why 16 of 40 respondents (40%) had inconsistent views: some condemned Hardin for his harshness yet would not sacrifice themselves. Others agreed with Hardin’s harsh approach, yet were willing to sacrifice, in a manner more consistent with Heilbroner. The specific variables considered by the various respondents in the given hypothetical situation may have outweighed the views they claimed to possess about Hardin’s harsh rationality.

It should be noted that when deciding whether to help other individuals, such decisions often include both a cognitive component and an emotional component (Fehr and Rockenbach 2004), and this also was reflected in the data, especially in the desire to avoid guilt and remorse. People tend to “evaluate the action in terms of its anticipated costs … and benefits … as well as its anticipated emotional consequences (how good will it make me feel to help/how bad will I feel if I do not help)” (Dora 2019). Even some of those that expressed that they would hold on to their retirement acknowledged that they did so at the expense of their own feelings (“I feel horrible [not being willing to give up my retirement]”). The emotional component present in tackling moral dilemmas and in determining whether or not to help must not be overlooked.

## Conclusion

Egoism and altruism often result in similar outcomes, and as Piccinini and Schulz (2018) point out, there is nothing morally problematic about this trend. An individual may display a helpful behavior, all the while acting out of self-interest, either consciously or unconsciously, with the result of such behavior being actual help to others. Even if pure classical psychological altruism does not exist and even if helpful actions are performed primarily out of self-interest, this does not negate actual, helpful outcomes. From an academic standpoint, scholars will continue to examine and debate self-interest and altruism. But from a more pragmatic viewpoint, as far as consequences and outcomes, the end results are often the same. Compassion is offered. Individuals are helped.

Clearly, formal ethics training is important. This is an empirical and observable fact. A study of ethics certainly enhances moral sensitivity and helps us to more easily recognize an ethical issue as an ethical issue (Des Jardin 2013). But it also prompts us to think about why we do what we do (Des Jardin 2013). Formal ethics training helps us to better understand ourselves, and the points of view and motivation of others as well. In this study, students were forced to recognize their own inconsistiencies and in some cases, to confront those inconsistencies and account for them. They were also made more aware of the sometimes inconsistent points-of-view of their classmates. The approach outlined in this study encourages reflective practice, a process whereby participants are able to examine their own assumptions across multiple perspectives. Reflective practice hinges on confronting previously unchallenged assumptions, thereby making personal transformation in the participants more likely (Brookfield 1998).

The Hardin and Heilbroner essays and the associated questions outlined here could be used across a variety of course offerings to spark discussion and to promote meaningful self-reflection and transformation. These essays and questions could also be used to argue for the importance of compassion, to reinforce the importance of personal responsibility, and to promote consistency, so that students do not become hardened towards their fellow human beings.

## Data Availability

Data and materials were obtained from the students of Dr. Bradley R. Reynolds, in a class entitled Values and the Environment. Informed Consent was obtained, and all procedures were carried out in accordance with UT Chattanooga’s Institutional Review Board for the Protection of Human Subjects. Raw data are currently in the possession of Dr. Reynolds.
